# TRIM proteins in hepatocellular carcinoma

**DOI:** 10.1186/s12929-022-00854-7

**Published:** 2022-09-13

**Authors:** Kan Lu, Yonglong Pan, Zhao Huang, Huifang Liang, Ze-yang Ding, Bixiang Zhang

**Affiliations:** 1 Hepatic Surgery Center, and Hubei Key Laboratory of Hepato-Pancreato-Biliary Diseases, Tongji Hospital, Tongji Medical College, Huazhong University of Science and Technology, Wuhan, 430030 Hubei China; 2 Clinical Medical Research Center of Hepatic Surgery at Hubei Province, Wuhan, Hubei China; 3 Key Laboratory of Organ Transplantation, Ministry of Education, Wuhan, China; 4 Key Laboratory of Organ Transplantation, National Health Commission, Wuhan, China; 5 Key Laboratory of Organ Transplantation, Chinese Academy of Medical Sciences, Wuhan, China

**Keywords:** Tripartite motif (TRIM), Hepatocellular carcinoma (HCC), Ubiquitination, E3 ubiquitin ligase

## Abstract

The tripartite motif (TRIM) protein family is a highly conserved group of E3 ligases with 77 members known in the human, most of which consist of a RING-finger domain, one or two B-box domains, and a coiled-coil domain. Generally, TRIM proteins function as E3 ligases to facilitate specific proteasomal degradation of target proteins. In addition, E3 ligase independent functions of TRIM protein were also reported. In hepatocellular carcinoma, expressions of TRIM proteins are both regulated by genetic and epigenetic mechanisms. TRIM proteins regulate multiple biological activities and signaling cascades. And TRIM proteins influence hallmarks of HCC. This review systematically demonstrates the versatile roles of TRIM proteins in HCC and helps us better understand the molecular mechanism of the development and progression of HCC.

**Supplementary Information:**

The online version contains supplementary material available at 10.1186/s12929-022-00854-7.

## Introduction

The tripartite motif (TRIM) protein family is a highly conserved group of RING-type E3 ligases with 77 members known in the human, most of which consist of a RING-finger domain, one or two B-box domains, and a coiled-coil domain [1]. Dysregulation of TRIM proteins has been found and shown crucial roles in different types of diseases including inflammation, viral infection, and cancer [2–4].

Liver cancer is the fourth leading cause of cancer-related death globally, and hepatocellular carcinoma (HCC) represents approximately 90% of primary liver cancer [5, 6]. In HCC, TRIM proteins have impacts on cell proliferation, apoptosis, cancer metastasis, metabolic reprogramming, stemness, carcinogenesis, immunogenicity, and resistance to cancer therapies. Furthermore, targeting TRIM proteins showed its potential effects on HCC. In this review, we summarize the roles of TRIM proteins in HCC as ubiquitin ligases or non-ubiquitination roles. We systematically demonstrate the biological functions of TRIM proteins in HCC and summarize the signaling cascades affected by TRIM proteins.

## Expression pattern and biological functions of TRIM proteins in HCC

### Structural classification and domain functions of TRIM proteins

**Fig. 1:**
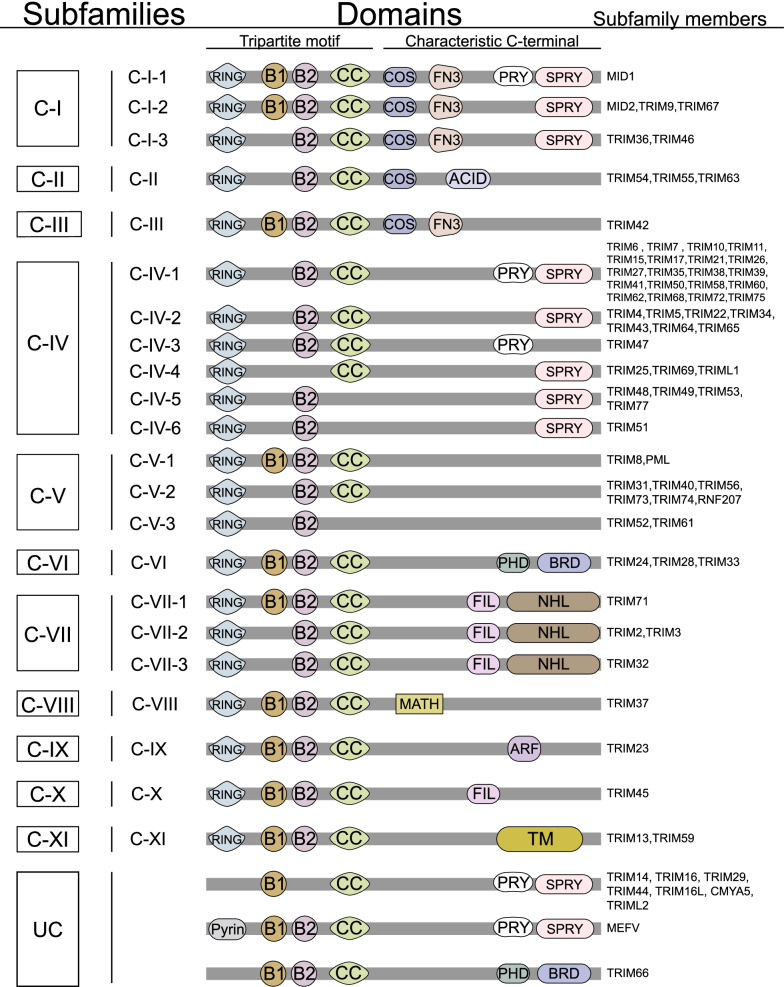
Structure of TRIM proteins. TRIM proteins are classified into subfamily C-I to C-XI according to different C-terminal domains, and a special subfamily UC without RING domain. List of abbreviation: COS domain (COS), Fibronectin type-III domain (FN3), PRY domain (PRY), B30.2/SPRY domain (SPRY), acid-rich region (ACID), filamin-type I domain (FIL), NHL domain, PHD domain, bromodomain (BRD), Meprin and TRAF-homology domain (MATH), ADP-ribosylation factor family domain (ARF), and transmembrane region (TM)

The tripartite motif (TRIM) protein family is named for their highly conserved RING domain, B-box domains, and the coiled-coil (CC) region at N-terminal. Unlike N-terminal domains, C-terminal domains of TRIM proteins vary in different subtypes, and TRIM proteins can be classified into subfamily C-I to C-XI according to distinctive C-terminal domains [1]. In detail, C-terminal domains of TRIM proteins including COS domain, Fibronectin type-III domain (FN3), PRY domain, B30.2/SPRY domain (SPRY), acid-rich region (ACID), filamin-type I domain (FIL), NHL domain, PHD domain, bromodomain (BRD), Meprin and TRAF-homology domain (MATH), ADP-ribosylation factor family domain (ARF), and transmembrane region (TM). Another subfamily called UC refers to 8 TRIM proteins without RING domain (Fig. 1).

Generally, TRIM proteins are E3 ubiquitin ligases based on their RING domain [7]. B-box domains contain one or two different zinc-binding motifs and are divided into 2 types. They promote the catalytic effect of RING or mediate ubiquitination of substrates independently [8, 9]. The Coiled-coil region executes a conserved scaffolding function of forming anti-parallel homo- or hetero-dimerization [10–12]. In detail, members of C-IV family (TRIM7, TRIM11, TRIM22, TRIM25, TRIM26, TRIM50, TRIM65, TRIM72), C-V family (TRIM31, TRIM52), C-VI family (TRIM28, TRIM33), C-VII family (TRIM71), C-XI family (TRIM59), UC family (TRIM14) exert their roles as E3 ligases in HCC (Table 1). TRIM proteins either promote or inhibit carcinogenesis and cancer progression mainly depending on specific identifying and degrading oncogenic or tumor suppressive proteins.

**Table 1:** Oncogenic/tumor-suppressive role of TRIM proteins in HCC

Name	Oncogenic or tumor suppressive	Effects	Mechanisms	References
TRIM3	Suppressive	↓ Proliferation ↓ Metastasis	G0/G1 cycle arrest	[87]
TRIM7	Oncogenic	↑ Proliferation	Activate DUSP6/p38 pathway	[43]
			Inactivate TP53/p21	
	Suppressive	↓ Proliferation ↓ Invasion	Inhibit SRC-mTORC1-S6K1 axis	[14]
			Down-regulate SRC	
TRIM10	–	–	Block interaction between IFNAR1 and TYK2	[15]
TRIM11	Oncogenic	↑ Proliferation ↑ Invasion ↑ Metastasis ↓ Autophagy	Down-regulate TP53	[85]
			Promote EMT Activate PI3K/AKT pathway	[103]
				[16]
			Inactivate AMPK pathway	[111]
TRIM16	Suppressive	↓ Invasion ↓ Metastasis	Suppress ZEB2 and EMT	[20]
PML	Oncogenic	↑Arsenic trioxide resistance ↑ Angiogenesis	Up-regulate ALDH3A1	[30]
			Metabolic reprogramming	[113]
	Suppressive	↓ Spontaneous HCC ↑ Apoptosis ↓ Liver steatosis	Strengthen DNA damage response and repair	[126]
			Upregulate TRAIL	[94]
			Suppress IL-6-induced STAT3 activation	[132]
			Transactivate P53	[84, 93]
			Inhibit cancer stem cells	[119]
			Co-activate Fas	[91]
TRIM22	Suppressive	↓ HBV	Inhibit core promoter of HBV	[13, 50]
TRIM23	Oncogenic	↑ Proliferation ↓ Apoptosis	Mediate IKKγ ubiquitination	[51]
TRIM24	Suppressive	↓ Spontaneous HCC ↓ Proliferation ↓ Invasion ↓ Metastasis	Attenuate retinoic acid receptor (RARα)	[117]
			Form regulatory complexes with TRIM28 and TRIM33	[74]
			Chromatin remodeling Inhibit VL30-ERV	[77]
			Inhibit STAT1	[76]
TRIM25	Oncogenic	↑ Proliferation ↓ Apoptosis ↑ Epirubicin resistance	Activate Keap1-Nrf2 pathway	[97]
			Activate PTEN/AKT pathway	[32]
			MAP3K13-TRIM25-FBXW7α-c-Myc protein axis	[88]
	Suppressive	↓ Metastasis ↓ HBV	IFN/IL-27/TRIM25/RIG-1 axis	[124]
			Down-regulate MTA1	[106, 107]
TRIM26	Oncogenic	↑ HCV	Down-regulate NS5B	[139]
TRIM28	Oncogenic	↑ Warburg effect	Down-regulate FBP1	[110]
	Suppressive	↓ Spontaneous HCC ↑ Sexual dimorphic metabolic syndrome	Epigenetic instability Inactivate AKT pathway Inactivate Wnt/β-catenin pathway	[72]
			ERK1/2-MAPK pathway	[112]
			Chromatin remodeling	[67, 71]
TRIM29	Suppressive	↓ Proliferation ↓ Invasion ↓ Metastasis	Inactivate Wnt/β-catenin pathway	[44]
TRIM31	Oncogenic	↓ Anoikis ↑ Proliferation ↑ Invasion ↓ Autophagy	Down-regulate TSC1–TSC2 to over-activate mTORC1 pathway	[34]
			Over-activate AMPK pathway	[99]
			Down-regulate TP53	
TRIM32	Oncogenic	↑ Proliferation ↑ Oxaliplatin resistance	Accelerate G1 transition	[35]
TRIM33	Oncogenic	↑ Tumor growth (early phase)	Down-regulate SMAD4 to inhibit Smad/TGF-β pathway	[22]
	Suppressive	↓ Spontaneous HCC ↓ Metastasis ↓ Immune escape	Down-regulate SMAD4 to inhibit Smad/TGF-β pathway	[22]
			circTRIM33-12	[59]
TRIM35	Suppressive	↓ Warburg effect	Inhibit PKM2 phosphorylation	[23, 109]
TRIM37	Oncogenic	↑ Metastasis ↑ Sorafenib resistance	Promote EMT	[36]
			Inactivate β-catenin pathway	
			Activate AKT pathway	[37]
TRIM44	Oncogenic	↑ Proliferation ↓ Apoptosis ↑ Invasion ↑ Migration ↑ Doxorubicin resistance	Accelerate G1/S transition	[38]
			Promote EMT	
			Activate NF-κB pathway	
			SPATS2/TRIM44/STAT3 axis	[105]
TRIM50	Suppressive	↓ Invasion ↓ Metastasis ↑ Anoikis	Down-regulate SNAIL	[24]
			Activate Wnt/β-catenin pathway	
TRIM52	Oncogenic	↑ Proliferation ↑ Invasion ↑ Metastasis	Down-regulate PPM1A	[39]
			Inactivate TP53	
TRIM55	Suppressive	↓ Invasion ↓ Metastasis	Down-regulate MMP2 to inhibit EMT	[25]
TRIM56	Suppressive	↑ Proliferation	Down-regulate RBM24	[26]
			Inactivate Wnt/β-catenin pathway	
TRIM59	Oncogenic	↑ Proliferation ↑ Invasion ↑ Metastasis	Down-regulate PPM1B	[40]
			Accelerate G1/S transition	
			Promote EMT	[86]
TRIM62	Oncogenic	↑ Sorafenib resistance ↑ Proliferation ↑ Invasion	Activate NF-κB pathway	[147]
TRIM65	Oncogenic	↑ Tumor growth ↑ Metastasis	HMGA1/TRIM65/Axin1/β-catenin axis	[83]
TRIM66	Oncogenic	↑ Proliferation ↑ Invasion ↑ Metastasis	Activate Wnt/β-catenin pathway	[101]
			Inhibit EMT	[41]
TRIM71	Oncogenic	↑ Proliferation	Inhibit p21 mRNA Non-canonical nonsense-mediated decay	[18]
			Down-regulate AGO2	[42]
TRIM72	Suppressive	↓ Sorafenib resistance	Inhibit RAC1-MAPK pathway	[146]

A summary of functions and specific mechanisms of TRIM proteins in HCC

↑: promoting; ↓: suppressing

N-terminal and C-terminal domains exert functions cooperatively or independently during the biological process in HCC. The SPRY helps the nuclear translocation of TRIM22 [13]. The SPRY also mediates the interaction of tyrosine-protein kinase Src (SRC) with TRIM7, the intracellular part of interferon alpha/beta receptor 1 (IFNAR1) with TRIM10, and pleckstrin homology domain leucine-rich repeats protein phosphatase 1 (PHLPP1) with TRIM11, to promote their substrate degradation [14–16]. Notably, TRIM14 has no RING domain, but it is still able to mediate ubiquitination degradation of NS5A through SPRY [17]. The NHL domain helps with the recognition between TRIM71 and a structural RNA stem-loop motif within the 3’-untranslated region (UTR) of CDKN1A mRNA [18]. Functions of other C-terminal domains need further research in HCC.

### Expression, mutation, and regulation of TRIM proteins in HCC

Expressions of TRIM proteins are frequently altered in HCC patients (Table 2). Researches show that TRIM3, TRIM16, TRIM26, TRIM33, TRIM35, TRIM50, TRIM55, TRIM56, and TRIM58 are low-expressed in HCC samples [19–27]. TRIM11, TRIM14, PML, TRIM25, TRIM28, TRIM31, TRIM32, TRIM37, TRIM44, TRIM52, TRIM59, TRIM66, and TRIM71 are high-expressed in HCC samples [28–42]. Interestingly, TRIM7 and TRIM29 exhibit opposite tendencies according to different studies. The difference may be the results of HBV infection status, tumor stages, or different detecting technology [14, 43–45].

**Table 2:** TRIM expression and related clinical characteristics

Name	Tendency in HCC	Case number	Prognosis indicator	Hazard ratio	p-value	Relative clinicopathological characteristics	References
TRIM3	Low	129	OS	0.562 (0.356–0.888)	0.014	Tumor size, histological grade, AFP, TNM stage	[19]
TRIM7	High	84	OS	–	0.0044	Tumor size, pTNM stage, serum HBV DNA copy number, and AFP	[43]
	Low	80					[14]
TRIM11	High	117	OS	2.98 (1.19–4.72)	< 0.01	AFP, pathological grade	[28]
			DFS	2.34 (0.92–3.26)	0.031		
TRIM14	High	166	OS	1.657 (1.031–2.687)	0.018	ALT, CRP, tumor size, tumor number, vascular invasion, BCLC stages, and TNM stages	[29]
			RFS	2.297 (1.184–2.312)	0.007		
TRIM16	Low	61	–	–	–		[20]
PML	High	40	OS		0.038	Age, HBsAg positive	[30]
			Cumulative relapse rate		0.01		
TRIM25	High	25	–	–	–		[32]
TRIM26	Low	242	OS	–	0.0265	AFP, American Joint Committee on Cancer (AJCC) T stage, Cancer Liver Italian Program (CLIP) stage	[21]
			RFS		0.0308		
TRIM28	High	116	OS	2.151 (1.032–4.486)	0.041	AFP, tumor size, tumor stage	[33]
TRIM29	High	90	OS	–	0.011		[45]
	LOW	20	–	–	–	Vascular invasion, Tumor differentiation	[44]
TRIM31	High	108			< 0.001	Tumor volumes, TNM stages, Edmenson grade	[34]
TRIM32	High	116	OS	0.523 (0.286–0.958)	< 0.01	Histological grade, tumor size, HBsAg positive	[35]
TRIM33	Low	204	OS	1.898 (1.101–3.268)	0.021	Tumor encapsulation, vascular invasion, differentiation, TNM stage, and BCLC stage	[22]
			Recurrence	1.751 (1.155–2.653)	0.008		
TRIM35	Low	688	OS	0.586 (0.376–0.913)	0.018		[23]
			Time to recurrence	0.643 (0.435–0.950)	0.027		
TRIM37	High	90	OS	–	0.029	Tumor size, tumor stage	[36]
	High	53	DFS		0.012		[37]
TRIM44	High	106	OS	3.442 (1.318–8.989)	0.012	Tumor size, vascular invasion, intrahepatic metastasis, distant metastasis	[38]
TRIM50	Low	79				TNM stages, BCLC stages, and metastasis	[24]
TRIM52	High	87				TNM stages, tumor number	[39]
TRIM55	Low	100	OS	0.425 (0.193–0.934)	0.033	vascular invasion, TNM stages	[25]
TRIM56	Low	41	OS/RFS	–	–	T stage	[26]
TRIM58	Low	43					[27]
TRIM59	High	103	OS		< 0.01	Tumor size	[40]
TRIM66	High	88				TNM stages	[41]
TRIM71	High	106	OS		0.008	AFP, tumor grade, tumor stage, early recurrence	[42]

Expression tendencies of TRIM proteins in HCC have been list. They are significantly associated with several clinical characteristics and may be independent prognostic indicators for HCC

*OS* overall survival; *RFS* recurrence-free survival; *AFP* α-fetoprotein; *CRP* C-reactive protein

Genetic alterations for TRIM proteins are common. We explore the genetic alteration events for TRIM proteins in 353 HCC patients through TCGA in the cbioportal [46, 47]. TRIMs are altered in 56% (196/353) of patients. TRIM11(7%), TRIM17(6%), TRIM35(6%), TRIM46(12%), TRIM55(7%), TRIM58(8%), TRIM67(7%), and TRIML1(5%) exhibit significantly higher mutation rate (Additional file 1: Fig. S1). TRIM proteins are also associated with common genetic mutations in HCC. We select top5 mutated genes according to TCGA-LIHC (TP53, TTN, CTNNB1, MUC16, and ALB) together with 10 genes impacting common pathways in HCC (AXIN1, APC, IRF2, CDKN2A, ARID1A, ARID2, KRAS, PIK3CA, RPS6KA3, NFE2L2) [48]. We explore the differential gene expression under different mutation statuses in HCC through TIMER2.0 [49]. The most linked genes are TP53(35/76), CTNNB1(25/76), and AXIN1(24/76) (Additional file 2: Table S1).

Apart from genetic variations, expressions of TRIM proteins are also modulated via epigenetic mechanisms, including DNA methylation, mi-RNA, circRNA, and long non-coding RNAs (lncRNA). TRIM21 is down-regulated by methylation in its 5′-UTR [50]. TRIM33 is reduced through aberrant CpG methylation at its promoter [22].

Mi-RNAs regulate gene expression by inhibiting transcription or inducing decay of mRNAs. Transcript of TRIM23 is targeted by miR-194, whose inhibition is a key process in NF-κB activation [51]. High-expressed miR-837 inhibits TRIM25 expression in HCC [52]. MiR-424-5p acts as a tumor suppressor by targeting TRIM29 [45]. MiR-29c-3p down-regulates TRIM31 expression [53]. MiR-4417 down-regulates TRIM35 [54]. And miR-4698 down-regulates TRIM59 [55]. Furthermore, circRNAs can act as sponges of miRNAs to repress miRNA function. Circ_0091579 acts as a sponge of miR-136-5p to up-regulate TRIM27 [56]. Circular RNA PVT1 (CircPVT1) acts as a sponge of miR-377 to up-regulate TRIM23 and promote HCC [57]. Hsa-circ-0026134 acts as a sponge of miR-127-5p to down-regulate TRIM25 [58]. Besides, tripartite motifs containing 33-derived circRNA (circTRIM33-12) acts as a sponge of miR-191 to up-regulate Methylcytosine dioxygenase TET1 (TET1) and prevents HCC progression [59].

LncRNA XIST directly targets miR-192 to up-regulate TRIM25 [60]. LncRNA rhophilin Rho GTPase binding protein 1 antisense RNA 1 (RHPN1-AS1) promotes TRIM16 expression [61]. LncRNA RP11-286H15.1 binds to poly(A) binding protein 4 (PABPC4) and promotes its ubiquitin degradation. while PABPC4 could enhance TRIM37 mRNA stabilization [62].

### Prognostic roles of TRIM proteins in HCC

Dysregulation of TRIM proteins significantly influences the prognosis of HCC. The hazard ratios (HR), case numbers, p-values, type of survival (overall survival (OS), recurrence-free survival (RFS)), and associated clinical characteristics are recruited in Table 2. We have also made a cox regression analysis for all TRIM proteins based on TCGA-LIHC patients with OS for more than a month (Additional file 2: Table S2) (Additional file 3: Fig. S2).

Besides, bioinformatic mining revealed that TRIM28, TRIM37, TRIM45, and TRIM59 could serve as efficient biomarkers in predicting OS, PFS, DSS, and RFS based on TCGA [63, 64]. Another study built a TRIM gene-based signature (including TRIM5, MID1(TRIM18), TRIM21, TRIM32, TRIM44, and TRIM47), which shows decent efficiency in predicting OS [HR: 6.630 (3.030–14.504), p < 0.001] in TCGA and GSE76427. A nomogram based on risk score, age, and TNM stage also showed better discrimination in predicting OS [65].

Furthermore, combinations of TRIM proteins with other proteins show higher efficiency in clinical assessments. Our study found that the combination of TRIM33 and phosphorylated SMAD2 is more efficient in predicting recurrence and OS in HCC [22]. TRIM28/minichromosomal maintenance complex component 6 (MCM6) is a novel marker for diagnosing HCC [66]. Zinc finger protein 354C (ZNF354C)/TRIM28/HDAC6 and TRIM35/pyruvate kinase isoform M2 (PKM2) are more effective prognostic factors for HCC [23, 67].

### TRIM proteins exert epigenetic regulations in HCC

#### Roles in chromatin remodeling

**Fig. 2:**
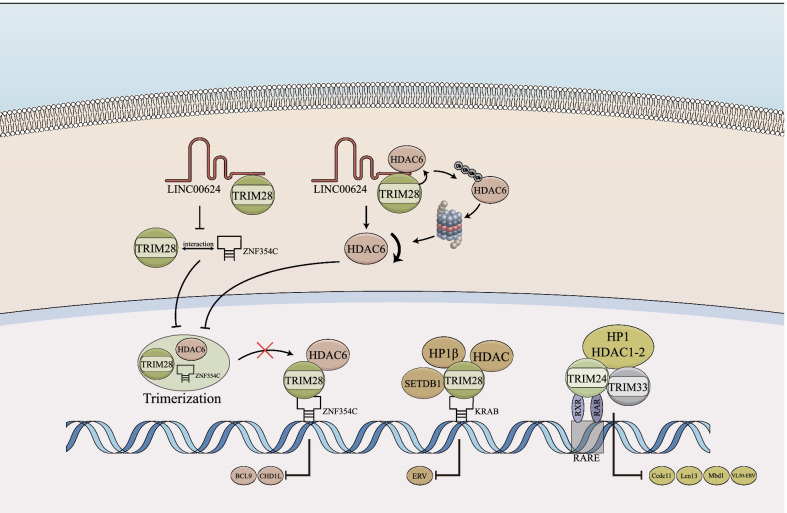
TRIM proteins in chromatin remodeling. TRIM28 is the scaffold for chromatin-remodeling complex, which comprises the SETDB1, HDACs, nuclear remodeling factors, and HP1. LINC00624 decrease the trimerization of TRIM28, HDAC6, and ZNF354C through hindering the interaction between TRIM28 and ZNF354C, and promoting TRIM28-mediated ubiquitin degradation of HDAC6. The dimers formed of TRIM24 and TRIM33 are recruited to the RARE at the LTR, which leads to the silencing of RA signaling or VL30 and prevents HCC. *SETDB1* SET domain bifurcated 1; *HDAC* histone deacetylases; *HP1* heterochromatin protein 1; *ZNF354C* Zinc finger protein 354C; *RARE* retinoic acid–responsive elements; *LTR* long terminal repeats; *RA* retinoic acid; *VL30* virus-like 30S-class ERVs

Members of the C-VI subfamily (TRIM24, TRIM28, and TRIM33) showed regulator effects on chromatin remodeling. TRIM28 is the scaffold of chromatin-remodeling complexes, consisting of the histone methyltransferase SET domain bifurcated 1 (SETDB1), histone deacetylases (HDACs), nuclear remodeling factors, and heterochromatin protein 1 (HP1) [68]. TRIM28 is recruited to the DNA via its interaction with Krüppel-associated box zinc finger proteins (KRAB) and synergistically inhibits KRAB-suppressed genes like endogenous retro-viruses (ERV) family [69, 70]. HP1, especially HP1β, mediates specific retrotransposons silent partly through TRIM28, whose dysregulation in KRAB/TRIM28/SETDB1 complex is determinant in HP1-dependent hepatic tumorigenesis (Fig. 2) [71]. LINC00624 promotes ubiquitin degradation of HDAC6 and interacts with TRIM28 to inhibit the conjugation between ZNF354C and TRIM28, therefore disrupting the HDAC6-TRIM28-ZNF354C corepressor complex formation and eliminating transcription suppression of Chromodomain-helicase-DNA-binding protein 1-like (CHD1L) and lymphoma 9-like protein (BCL9) [67]. Aging and obesity strengthen TRIM28-dependent epigenetic regulation, such as activating tumor-associated molecular patterns (TAMPs), dampening the farnesoid x receptor (FXR) pathway, over-activating β-catenin, and altering the androgen pathway [72]. Besides, SETDB1 and its cofactor TRIM28 physically interact with METTL3 (methyltransferase-like 3) in mouse embryonic stem cells, which shows TRIM proteins may participate in N^6^-methyladenosine (m^6^A) methylation of mRNA [73].

Similarly, TRIM24 and TRIM33 form chromatin remodeling complexes with HP1 and HDAC (Fig. 2) [74, 75]. TRIM24 and TRIM33 are recruited to the retinoic acid-responsive elements (RARE) at the long terminal repeats (LTRs) to inhibit the expression of RA receptor α (RARα)-mediated transcription, signal transducer, activator of transcription 1 (STAT1), or virus-like 30S-class ERVs (VL30) [76, 77]. Activation of the VL30 elicits chronic inflammation and generates enhancer RNAs to promote transcription of TRIM24 and neighboring genes [77]. Interestingly, the KRAB-zinc finger proteins (ZFPs)-TRIM28 system could also affect VL30 in mouse embryonic fibroblasts [78]. It is worth further investigating whether TRIM28 regulates VL30 synergistically with TRIM24 and TRIM33 in HCC.

#### Roles in mRNA instability

TRIM71, a member of the C-VII subfamily, disrupts miRNA-mediated gene silencing through mediating ubiquitin degradation of proteins argonaute (Ago) 1 and Ago2 to decrease RNA-induced silencing complex (RISC). Thus, TRIM71 inhibits functions of tumor-suppressive miR-let-7 and oncogenic miR-21 in HCC [42, 79]. However, a recent study contradicted that TRIM71 proceeds degradations of mRNA through non-canonical nonsense-mediated decay (NMD) rather than interfering RISC. TRIM71 destabilizes p21 mRNA through cooperating with NMD factors serine/threonine-protein kinase SMG1 (SMG1), SMG7, and regulator of nonsense transcripts 1 (UPF1) [18].

## Effects of TRIM proteins on hallmarks of carcinogenesis

### Sustaining proliferation

Sustaining proliferation is a hallmark of cancer. Aberrant expression of TRIM proteins leads to abnormal cell cycle progression and sustaining proliferation.

Our previous study revealed that TRIM33 suppresses the TGF-β pathway by mediating ubiquitin degradation of mothers against decapentaplegic homolog 4 (Smad4) and inhibiting the Smad2/3/4 complex formation. TRIM33 suppressed TGF-β down-stream p21, p15, and restored c-myc. Therefore, TRIM33 promotes proliferation in early-phase HCC [22]. TRIM52 mediates ubiquitin degradation of protein phosphatase, Mg^2+^/Mn^2+^ dependent 1A (PPM1A), which dephosphorylates Smad2 and Smad3 to inhibit the TGF-β pathway and results in increased expression of TP53 and p21 [39].

Cell cycle defect is a common feature of human cancers, whose central part is the harmonious control of cyclin-dependent kinases (CDKs), cyclin-dependent kinase inhibitors (CKIs), and cyclins [80, 81]. TRIM proteins regulate certain CDKs and CKIs to induce G1/S cell cycle arrest in HCC. TRIM28 interacts with Ubiquitin-conjugating enzyme E2 S (UBE2S) in the nucleus. They enhance p27 ubiquitination to mediate G1/S arrest, which can be blocked by cephalomannine [82]. TRIM65 interacts with AXIN1 and promotes ubiquitin degradation of AXIN1 to activate the β-catenin pathway and promote expressions of cyclin D1 and c-myc. TRIM65 is up-regulated by HMGA1 [83]. And TRIM71 suppresses functions of p21 mRNA through NMD [18]. P53 is mainly activated by the PML-IV isoform in PML-nuclear bodies (PML-NBs) [84]. TRIM11 is associated with decreased TP53, p21, p27 expression and increased cyclin D1 expression [85]. TRIM59 promotes ubiquitin degradation of protein phosphatase 1B (PPM1B), which dephosphorylates and inactivates CDK2. TRIM59 also decreases cyclin E1, CDK6, and CDK1, and degrades TP53 [40, 86]. TRIM7 facilitates ubiquitin degradation of Dual specificity protein phosphatase 6 (DUSP6) to inhibit downstream inactivation of p38 and MAPK, and TRIM7 is associated with high TP53 and p21 expressions [43]. In addition, TRIM3 induces G0/G1 cycle arrest [87]. Associations between TRIM and other phases of cell cycle in HCC need further research.

Mutation of myc is significantly deleterious to HCC development, abnormal activation of myc-related signaling is crucial for the proliferation of HCC. Mitogen-activated protein kinase kinase kinase 13 (MAP3K13) promotes phosphorylation and suppresses proteasomal degradation of TRIM25. TRIM25 mediates the ubiquitin degradation of F-box/WD repeat-containing protein 7α (FBXW7α), which is the main E3 ligase that down-regulates c-myc [88]. TRIM56 is associated with up-regulated c-Myc and activated β-catenin [26]. TRIM71 inhibits functions of miR-let-7 and up-regulated down-stream c-Myc, Lin-28B, HMGA2 and type 1 insulin-like growth factor receptor (IGF1R) [42].

### Resistant to apoptosis

Apoptosis is a process of programmed cell death that can be triggered by either the extrinsic or the intrinsic pathways [89]. PML induces apoptosis in parvovirus H-1 or HCV-infected HCC cells [90, 91]. PML induces apoptosis through P53, Fas, TNF, tumor necrosis factor-related apoptosis-inducing ligand (TRAIL), and caspase pathways [91–94]. Knockdown of TRIM44 decreases the expression of cellular inhibitors of apoptosis 1 (c-IAP1), c-IAP2, and XIAP, which are anti-apoptosis targets in the NF-κB signaling pathway [38, 95].

Abnormal antioxidative response via aberrant Kelch-like ECH-associated protein 1 (Keap1)- nuclear factor erythroid 2-related factor 2 (NRF2) signaling is a common event in the progression of HCC [96]. TRIM25 promotes ubiquitin degradation of Keap1, therefore over-activating NRF2 to alleviate oxidative stress and reduce apoptosis in HCC [97].

Anoikis is defined as the detached from the extracellular matrix (ECM) -induced apoptosis, and resistance to anoikis is a hallmark of cancer [98]. TRIM31 targets TP53 proteasomal degradation to over-activates the AMP-activated protein kinase (AMPK) pathway to promote anoikis [99]. Oppositely, TRIM50 down-regulates SNAIL through ubiquitin degradation, therefore reverses EMT and inhibits anoikis resistance [24].

### Metastasis of HCC

The dysregulated Wnt/β-catenin pathway is a common event in HCC tumorigenesis and is related to stemness and aggressive phenotype of HCC [100]. TRIM66 facilitates glycogen synthase kinase-3 beta (GSK-3β) phosphorylation and thereby inhibits the β-catenin pathway [101]. Inversely, TRIM37 strengthens pS9GSK-3β expression, which is the inactive type of GSK3β [36]. TRIM29 inhibits the expression and phosphorylation of β-catenin to prevent metastasis [44, 45]. TRIM28 inhibits the activation of β-catenin triggered by aging and obesity [72]. TRIM65 and TRIM37 suppress metastasis through activating the β-catenin pathway [36, 62, 83].

Epithelial–mesenchymal transition (EMT) refers to the process that epithelial cells acquire mesenchymal features, which promotes invasion and metastasis in cancer [102]. TRIM11 interacts with PHLPP1 by SPRY domain and mediates proteasomal degradation of PHLPP1. Thereby it promotes the phosphorylation and activation of AKT. Thus, TRIM11 significantly promotes migration, invasion, and EMT [16, 103]. TRIM33 inhibits down-stream targets of TGF-β pathway, including MMP1, CXCR4, Snail, and Slug, to promote invasion and metastasis in end-phase HCC [22]. TRIM50 mediates ubiquitin degradation of SNAIL to reverse EMT [24]. TRIM16 suppresses zinc finger E-box-binding homeobox 2 (ZEB2) expression to down-regulate E-cadherin [20].

MMP proteins facilitate the degradation of ECM proteins. MMP2 and MMP9 are major MMPs in the pathogenesis of EMT in HCC [104]. TRIM55 is associated decreases expression of MMP2 and vimentin [25]. TRIM66 decreases MMP9 expression [41]. Spermatogenesis associated serine rich 2 (SPATS2) promotes HCC progression via the TRIM44-p-STAT3 axis. TRIM44 is associated with expressions of hypoxia-inducible factor 1α (HIF-1α), MMP9, serine/threonine-protein kinase pim-1 (PIM-1), and BCL-2 [105].

TRIM25 mediates ubiquitination of metastasis associated 1 protein (MTA1) at K98 to suppress metastasis in HCC [106, 107].

TRIM7 interacts with SRC and affects the SRC-mTORC1-ribosomal protein S6 kinase beta-1 (S6K1) axis [14]. TRIM31 mediates ubiquitin degradation of the TSC1-TSC2 complex to over-activate mTORC1 [34]. Therefore, TRIM7 and TRIM31 result in unresolved ER stress, autophagy suppression, and invasion.

### Metabolic reprogramming of HCC cells

The Warburg effect refers to the metabolic reprogramming in cancer that energy is generated through aerobic glycolysis instead of mitochondrial oxidative phosphorylation [108]. PKM2 is a rate-limiting enzyme in glycolysis, whose phosphorylation provides extra metabolic advantages for HCC cells. TRIM35 competes with fibroblast growth factor receptor 1 (FGFR1) to interact with PKM2 and consequently inhibits the phosphorylation of PKM2 [23, 109]. TRIM28/melanoma-associated antigen (MAGE)-A3/MAGE-C2 complex promotes the Warburg effect through ubiquitin degradation of fructose-1,6-bisphosphatase 1 (FBP1), which is the rate-limiting enzyme in gluconeogenesis [110]. TRIM11 is significantly induced upon glucose deprivation. TRIM11 down-regulates AMPKβ2 through ubiquitin degradation to suppress AMPK pathway and leads to starvation-induced autophagy [111].

Furthermore, TRIM proteins are involved in lipid, hormone, and biliary acid metabolism. Liver-specific TRIM28-knockout mice exhibit aberrant androgen receptor stimulation, biliary acid disturbances, and significantly altered gut microbiota such as *Prevotella, Akkermansia muciniphila, and Bacteroides uniformis,* which are species predominantly associated with metabolic dysfunction and inflammation. Notably, this abnormality can be completely abolished under axenic conditions [72]. Liver-specific TRIM28-knockout results in sexual dimorphic metabolic syndrome through activating the ERK1/2-MAPK pathway. Loss of TRIM28-dependent epigenetic silencing results in activation of fat-specific protein 27 (FSP27), glutathione S-transferase, Cyp2d9, Cyp2a, Cyp2b, and Cyp3a gene clusters, and thereby leads to male-predominant liver steatosis and adenoma [112].

PML-knockout mice show increased white fat initially, but exhibit weight loss and white fat browning in end-stage HCC with the metabolic reprogramming from glycogen storage to lipolysis [113]. PML-deficient HBsAg-transgenic mice showed obvious oxidative phosphorylation and fatty acid metabolism impairments and encountered early steatosis-specific liver tumorigenesis [114].

Besides, TRIM8 promotes phosphorylation of TGF-β–activated kinase 1 (TAK1) [115]. TRIM16 down-regulates phosphorylated TAK1 [116]. They regulate downstream c-Jun N-terminal kinase/p38 in hepatocytes and influence steatohepatitis progression, which may point out future studies in HCC.

### Initiation of HCC

Somatic hepatocyte-specific inactivation of TRIM24, TRIM28, or TRIM33 all promotes spontaneous HCC [74, 117]. TRIM24 forms quantities of dimers with TRIM33, and a few trimers with TRIM28 and TRIM33. Liver TRIM24-knockout induced HCC is significantly promoted by further loss of TRIM33, and is slightly hindered by further loss of TRIM28 [74]. Mechanistically, TRIM24 attenuates RARα-mediated transcription through chromatin remodeling as mentioned above. Thus, TRIM24 deficiency activates downstream targets of the RA pathway such as Cyp26a1, protein-glutamine gamma-glutamyl transferase 2 (TGM2), RBP1, and receptors for retinol uptake STRA6 (STRA6). Notably, deletion of a single allele of RARα is sufficient to restore the phenotype of TRIM24-knockout mice [117]. TRIM24 also binds to the RARE of STAT1 promoter to inhibit STAT1 expression and promotes expressions of tumor-suppressive factors such as p21, Bmyc, and hepatocyte nuclear factor 6 (HNF6) [76].

Tumor initiation cells (TICs) in HCC are a subset of HCC cells with stem cell features and influence the initiation, cell growth, drug resistance, and recurrence of tumors [118]. Arsenite treatment represses PML expression, which down-regulates Oct4, Sox2, and Klf4 expressions. As a result, it reduces viability and stemness of CD133+ CD13+ TICs and enhances sensitivity to pirarubicin in HCC [119].

### Immune responses

#### Anti-HBV effect

HBV- and HCV infection are leading risk factors for HCC worldwide, and dysregulated responses to the infection of HBV or HCV fuel the progression of HCC [120–122].

**Fig. 3:**
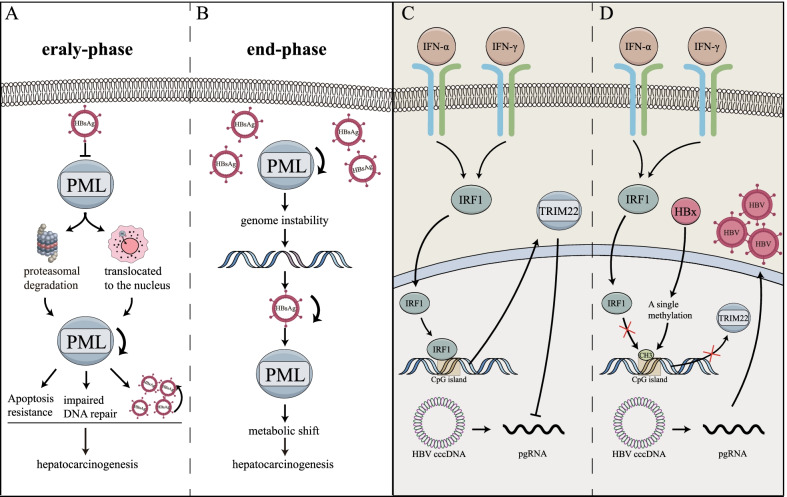
TRIM proteins in anti-HBV response of HCC. **A** PML is negatively correlated with HBsAg at the early-phase, because of proteasomal degradation and translocation to the nuclear. Down-regulated PML promotes apoptosis resistance and impairs DNA repair in HCC. **B** Long-term suppressed PML results in genome instability, which may confront the loss of HBV genes and HBsAg expression. However, PML turns out to be oncogenic since it enhances metabolic shift from glycogen storage to lipolysis. **C** TRIM22 is up-regulated under IFN stimulations. IFN activate IRF1, which is transferred to the nucleus and promote TRIM22 transcription by conjugating to its CpG island. **D** HBx suppresses IFN-induced transcription of TRIM22 gene through a single CpG methylation in its 5′-UTR, which reduces the IRF1 binding, thereby suppressing the IFN-stimulated induction of TRIM22 and exhibiting HBV immune escape. *IRF1* IFN regulatory factor-1; *HBx* HBV regulatory protein X; *UTR* untranslated region

Multiple TRIM proteins are identified inhibiting HBV replication in HCC, including TRIM5, TRIM6, TRIM11, TRIM14, TRIM25, TRIM26, TRIM31, and TRIM41 [123]. TRIM25 is an interferon-stimulated gene (ISG) augmented by IFN and IL-27, which mediate lysine 63-ubiquitination of RIG-I to suppress HBV replication [124]. PML is significantly associated with genomic instability and DNA repair in HBV-related HCC [125, 126]. PML is negatively correlated with HBsAg because of proteasomal degradation or translocation of HBsAg to the nucleus [113, 114, 125, 126]. PML suppresses the early phase of HCC since it enhances DNA repair and induces resistance to IFN-α or DNA damage-induced apoptosis (Fig. 3A), but turns out to be oncogenic in the end stage. It enhances a metabolic shift from glycogen storage to lipolysis, which implicates more energy available for driving HCC progression (Fig. 3B) [113, 125].

HBV regulatory protein X (HBx) stimulates HBV gene expression from the covalently closed circular (cccDNA) and is involved in HCC development [127]. TRIM14 is a STAT1-dependent type-I ISG. The TRIM14 SPRY domain interacts with the C-terminal of HBx to inhibit the formation of Smc-HBx-damage-specific DNA-binding protein 1 (DDB1) complex [128]. TRIM5γ is another type-I ISG which mediates HBx ubiquitin degradation through the B-box domain. TRIM31 is recruited by TRIM5γ and can also mediate HBx ubiquitin degradation [129]. Another study supplemented that TRIM31 is a type-III ISG and can be induced upon HBV replication [130].

HBx also regulates TRIM expressions. TRIM22 can be strongly stimulated by IFN-α and IFN-γ through IFN regulatory factor-1 (IRF1) in HCC. TRIM22 suppresses HBV core promoter by its nuclear-located RING domain, whose translocation is mediated by the SPRY domain (Fig. 3C) [13]. However, HBx protein down-regulates the transcription of TRIM22 through a single CpG methylation in its 5’-UTR to inhibit the binding between the promoter and IRF1, thereby inhibiting the IFN-stimulated induction of TRIM22 and resulting in HCC (Fig. 3D) [50]. And HBx protein promotes expressions of TRIM7 or TRIM52 [43, 131].

Besides, TRIM proteins may regulate STAT3 to regulate IFN responses. PML suppresses interleukin (IL)-6-induced Tyr705 and Ser727 phosphorylation of STAT3 and interferes with the interaction between STAT3 and HDAC3 to suppress the IL-6/STAT [132]. TRIM10 inhibits the association between non-receptor tyrosine-protein kinase TYK2 (TYK2) and IFNAR1 to inhibit the IFN/JAK/STAT pathway [15].

#### Anti-HCV effect

PML declines in HCV transgenic mice and develops more spontaneous or phenobarbital/diethylnitrosamine (DEN) induced HCC via down-regulating RNA NLRP12, Ras association domain-containing protein 6 (RASSF6), and TRAIL expressions [133–135]. HCV core protein interacts and inactivates PML-IV in PML-NBs to inhibit phosphorylation and acetylation of P53, which leads to dysregulation of Fas in HCC [91, 136]. IFN-α can compensate for the expression of PML suppressed by HCV core protein [94]. TRIM14 is an ISG and inhibits HCV infection by SPRY domain-dependent targeted degradation of NS5A protein [17, 137]. Similarly, TRIM22 mediates ubiquitin degradation of NS5A under IFN-α treatment to inhibit HCV replication [138]. TRIM26 expedites HCV replication in HCC through its interaction with HCV-encoded NS5B and mediating K27-linked ubiquitination of NS5B to promote NS5B-NS5A interaction [139].

#### Tumor microenvironment (TME)

**Fig. 4:**
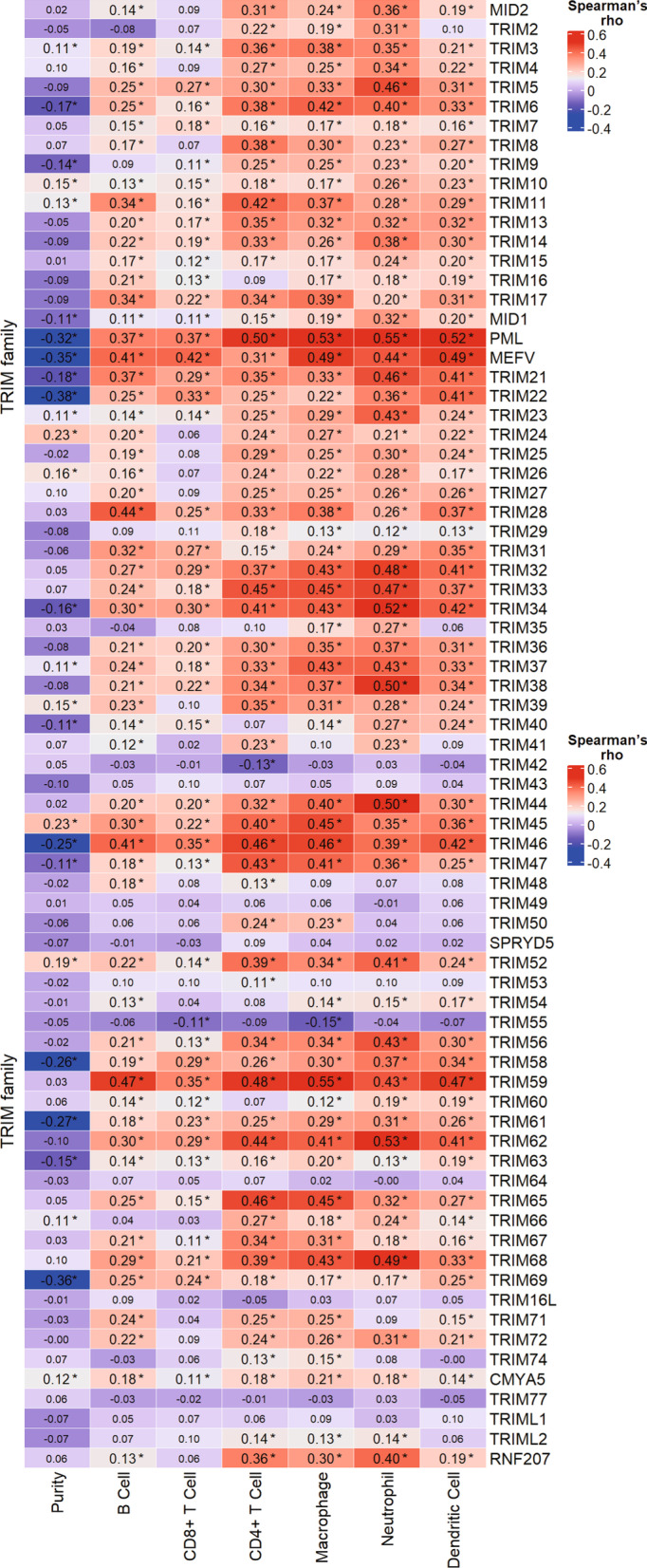
Correlation between TRIM and immune infiltrates in LIHC. Correlation between TRIM and abundance of 6 immune infiltrates in LIHC using TIMER database. The purity-corrected partial Spearman’s rho values are displayed with statistical significance (p < 0.05) marked with *. TRIM59, PML(TRIM19), TRIM46, and MEFV(TRIM20) significantly affect these immune infiltrations

The TME exists abundant of tumor cells as well as innate and adaptive immune cells, stromal cells, endothelial cells, and cancer-associated fibroblasts. Exploring of TME may help develop the treatment strategies in HCC [140]. We collected the associations between TRIM family and Immune Infiltrates in TIMER database (Fig. 4) [141, 142]. TRIM59, PML(TRIM19), TRIM46, and MEFV (TRIM20) significantly affect these immune infiltrations.

TRIM28 activated by Receptor-interacting serine/threonine-protein kinase 3 (RIPK3) loses its chromatin binding ability. Thus, RIPK3 transactivates NF-κB and SOX9, strengthens CD8+ T cell and DC maturation [143]. TRIM28 and SETDB1 form regulatory complex, whose loss could activate cGAS–STING innate immunity to strengthen antitumor effects of anti–PD-L1 [144]. Verteporfin inhibits PD-L1 through the STAT1-IRF1-TRIM28 signaling axis [145]. However, none of these results focus on HCC.

### Resistance to cancer therapies

Sorafenib is recommended as first/second line systematic therapy for BCLC B or C stage HCC patients [6]. TRIM72 interacts with Ras-related C3 botulinum toxin substrate 1 (RAC1) with its Coiled-coil domain. It down-regulates RAC1 through ubiquitin degradation and inhibits the RAC1-MAPK pathway to enhance sensitivity to sorafenib [146]. TRIM62 promotes phosphorylation of IKKβ and NF-κB p65 nuclear translocation. Therefore, TRIM62 activates the NF-κB pathway and induces sorafenib resistance [147]. TRIM37 activates the AKT pathway to induce sorafenib resistance [37].

Besides, TRIM25 strengthens sensitivity to epirubicin through promoting ubiquitin degradation of PTEN [32]. TRIM32 induces oxaliplatin resistance [35]. High-expressed TRIM44 accelerates doxorubicin resistance by activating the NF-κB pathway [38]. Arsenic trioxide (ATO) is a traditional chemotherapeutic drug for HCC patients [148]. ATO suppresses HCC formation synergistically with PML through promoting TP53, Bcl-2, and strengthening PML-NBs expression and functions [149]. However, PML also down-regulates the aldehyde dehydrogenase family 3 member A1 (ALDH3A1) by physically combining with the promoter to induce ATO resistance [30].

## Prospect

Currently, the poor prognosis and low percentage of patients responding to systemic therapies are characteristics of HCC, and new therapeutic methods for targeting HCC are urgently needed. As TRIM proteins exert functions mainly through the ubiquitin system (UPS), it seems feasible that use proteasomal inhibitors to block TRIM proteins to ameliorate HCC. Proteasome inhibitors like bortezomib, ixazomib, and carfilzomib have shown effectiveness in some cancer, but their applications are unsatisfying in HCC, as bortezomib in HCC in phase II trial lacked activity [150–152]. Carfilzomib and gankyrin inhibitors are far from clinical applications [153]. Several factors account for the ineffectiveness in common. Bortezomib may not inhibit the UPS in the liver as expected, or the dose and schedule need further modulations. Alternatively, the crosstalk of intertwined signaling pathways may counteract each other. Side effects of proteasome inhibitors like neuropathy also restrain their application [154].

Recently, proteolysis-targeting chimeras (PROTACs) give novel insight into applications of TRIM proteins. PROTACs technology employs E3 ligase ligands and fuses target protein with E3 ligase by a flexible chemical bond, to elicit ectopic ubiquitination and degrade specific target proteins [155]. PROTACs entered clinical research for cancer therapies in 2019 [156]. And the first oral PROTAC ARV-110 has shown effectiveness in prostate cancer [157]. TRIM proteins have promising applications in HCC through two aspects of PROTACs. TRIM proteins can be direct targets of PROTACs. For instance, dTRIM24 can recruit VHL E3-ligase to elicit potent and selective degradation of TRIM24 [158]. And dTRIM24 has successfully degraded TRIM24 in human metaplastic breast cancer patient-derived xenografts to decrease tumor cell viability [159]. TRIM proteins may also become mediums in PROTACs, which means recruiting TRIM proteins to specifically down-regulate some oncoproteins to alleviate HCC. But the design of new PROTACs ligand compounds is challenging since they need to conjugate the “right binding site” for limited ubiquitin sites as well as for reserving enough space to elongate the ubiquitin chain.

Notably, many virus proteins enable to hijack host E3 ligases to antagonize anti-viral factors, which may enlighten the development of PROTACs of TRIM proteins. These proteins seem natural and prototypical PROTACs [156, 160]. The HPV E6 oncoprotein employs ubiquitin-specific protease 15 (USP15) to degrade TRIM25 [161]. Murine gamma herpesvirus 68 induces proteasomal-dependent degradation of PML by the virion tegument protein ORF75c [162].

In addition, the C-VI subfamily (TRIM24, TRIM28, and TRIM33) might be the best transitional therapeutic targets in the future. This subfamily has powerful influences on the progression of HCC. Homozygous deletion of any of them leads to spontaneous HCC, and they regulate epigenetic silencing through chromatin remodeling. They affect diverse signaling pathways including the RA pathway, β-catenin pathway, TGF-β pathway, et.al. On the other hand, the PROTAC dTRIM24 has been invented. RA may also restrain the function of TRIM24. This may also enlighten our next-step transitional research.

Besides, there are still some unsolved problems in the TRIM family. The distribution is tightly linked to protein functions. We collect the intracellular location of TRIM proteins from UniProt (Additional file 2: Table S3) [163]. But few researchers concerning about the sublocation of TRIM in HCC. Another shortness is the relationship between TRIM and first- or second-line therapy drugs for HCC. It is worth further investigating whether TRIM may benefit our current therapies in HCC. As lncRNAs are essential in regulating expressions and functions of TRIM proteins, it seems better to investigate relationships between lncRNA and TRIM on m6A regulations or chemotherapy resistance in HCC.

## Conclusion

**Fig. 5:**
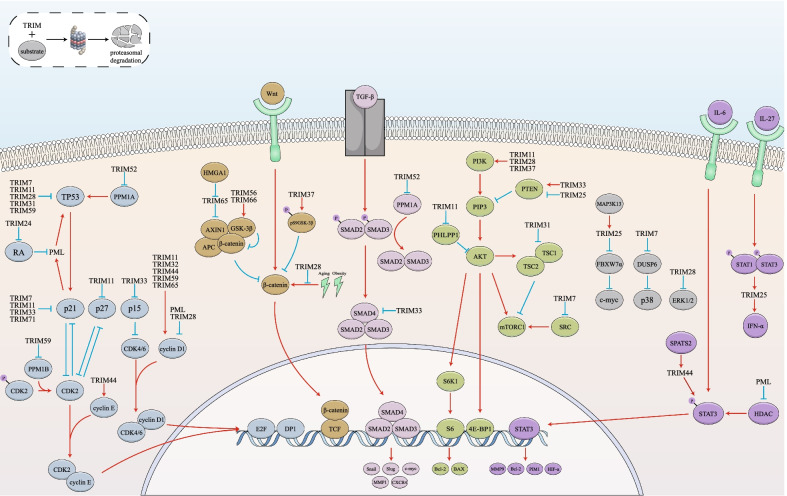
Signaling pathways that regulated by TRIM proteins. TRIM proteins affect multiple signaling cascades, including TGF-β pathway, cell cycle pathway, AKT/mTOR pathway, Wnt/β-catenin pathway, MAPK pathway, IFN/STAT pathway, and RA pathway. They mediate ubiquitin degradation of key proteins in pathways to activate or inactivate these signaling pathways

Growing clinical research has revealed that expressions of TRIM proteins are frequently altered and significantly associated with clinical indexes and prognosis in HCC. Some TRIM proteins are novel tumor markers and independent prognostic factors for HCC, indicating their potential in early diagnoses, prognosis assessments, and clinical therapies. In HCC, TRIM proteins regulate their proliferation, apoptosis, metastasis, metabolic reprogramming, immune responses, and resistance to cancer therapies. Mechanistically, TRIM proteins regulate levels and functions of downstream proteins through ubiquitination-dependent and independent mechanisms, and specific members of TRIM proteins regulate the activity of TGF-β/Smad, MAPK, PI3K-AKT, Wnt/β-catenin, cell cycle, STATs, and RA signaling cascades in HCC (Fig. 5). Targeting TRIM proteins showed therapeutic potential in HCC.

## Supplementary Information

**Additional file 1: Fig. S1.** Oncoprint plot of all somatic mutations of TRIM proteins in TCGA-LIHC using cbioportal database.

**Additional file 2: Table S1.** Differential gene expression under different mutation status in HCC through TIMER2.0. The logFC with statistical significance (p<0.05) are colored in the table. **Table S2.** Cox analysis for all TRIM proteins based on TCGA-LIHC patients with OS more than a month. **Table S3.** Intracellular location of TRIM proteins from uniprot.

**Additional file 3: Fig. S2.** A univariate cox analysis of every 75 TRIM proteins based on TCGA-LIHC dataset with p < 0.05.

## Data Availability

The datasets used and/or analyzed during the current study are available from the corresponding author upon reasonable request.

## Abbreviations

HCC: Hepatocellular carcinoma
TRIM: Tripartite motif
AKT: Protein kinase B
mTOR: Mammalian target of rapamycin
MAPK: MAP kinase
RING: RING-finger domain
B1 or B2: B-box domain
CC: Coiled-coil region
COS: COS domain
FN3: Fibronectin type-III domain
PRY: PRY domain
SPRY: B30.2/SPRY domain
ACID: Acid-rich region
FIL: Filamin-type I G domain
NHL: NHL domain
PHD: PHD domain
BRD: Bromodomain
MATH: Meprin and TRAF-homology domain
ARF: ADP-ribosylation factor family domain
TM: Transmembrane region
SRC: Proto-oncogene tyrosine-protein kinase Src
IFNAR1: Interferon alpha/beta receptor 1
IFN: Interferon
CDKN1A: P21
SETDB1: Histone methyltransferase SET domain bifurcated 1
HDAC: Histone deacetylase
HP1: Heterochromatin protein 1
KRAB: Krüppel-associated box zinc finger proteins
ERV: Endogenous retro-viruses
ZNF354C: Zinc finger protein 354C
BCL9: B-cell CLL/lymphoma 9 protein
CHD1L: Chromo-domain-helicase-DNA-binding protein 1-like
FSP27: Fat-specific protein 27
Cyp: Cytochrome p450
TAMPs: Tumor-associated molecular patterns
FXR: Dampening the farnesoid x receptor
LTRs: Long terminal repeats
RARE: Retinoic acid–responsive elements
RA: Retinoic acid
STAT: Signal transducer and activator of transcription
VL30: Virus-like 30S ERV
ZFP: Zinc finger proteins
miRNA: MicroRNA
AGO1: Protein argonaute
RISC: RNA-induced silencing complex
IGF1R: Type 1 insulin-like growth factor receptor
NMD: Nonsense-mediated decay
PTC: Premature termination codon
UPF1: Nonsense transcripts 1
UTR: Untranslated region
SMAD: Mothers against decapentaplegic homolog
PPM1A: Protein phosphatase, Mg^2^^+^/Mn^2^^+^ dependent 1A
PI3K: Phosphoinositide 3-kinase
PTEN: Phosphatidylinositol 3,4,5-trisphosphate 3-phosphatase and dual-specificity protein phosphatase PTEN
PHLPP1: Pleckstrin homology domain leucine-rich repeats protein phosphatase 1
TSC1: Hamartin
TSC2: Tuberin
S6K1: Ribosomal protein S6 kinase beta-1
GSK-3β: Glycogen synthase kinase-3 beta
MMP: Matrix metalloproteinase
RBM24: RNA-binding protein 24
HMGA1: High mobility group A1
CDKs: Cyclin-dependent kinases
CKI: Cyclin-dependent kinase inhibitors
UBE2S: Ubiquitin-conjugating enzyme E2 S
PML: TRIM19, promyelocytic leukemia
PML-NBs: PML-nuclear bodies
PPM1B: Protein phosphatase 1B
ERK: Extracellular signal-related kinases
MAP3K13: Mitogen-activated protein kinase kinase kinase 13
FBXW7α: F-box/WD repeat-containing protein 7α
DUSPs: Dual specificity phosphatases
ROS: Reactive oxygen species
Keap1: Kelch-like ECH-associated protein 1
Nrf2: Nuclear factor erythroid 2-related factor 2
RARα: Retinoic acid receptor α
HNF6: Hepatocyte nuclear factor 6
IL: Interleukin
SPATS2: Spermatogenesis associated serine rich 2
HIF-1α: Hypoxia-inducible factor 1α
PIM-1: Serine/threonine-protein kinase pim-1
TYK2: Non-receptor tyrosine-protein kinase TYK2
OS: Overall survival
DFS: Disease-free survival
TTR: The median time to recurrence
AFP: α-Fetoprotein
MCM6: Minichromosomal maintenance complex component 6
PKM2: Pyruvate kinase isoform M2
p27: Cyclin-dependent kinase inhibitor 1B
p15/ink4b: Cyclin-dependent kinase 4 inhibitor B
LncRNA: Long non-coding RNA
PABPC4: Poly(A) binding protein 4
Lin-28B: Protein lin-28 homolog B
IGF1R: Insulin-like growth factor 1 receptor
TRAIL: Tumor necrosis factor-related apoptosis-inducing ligand
AMPK: Monophosphate-activated protein kinase
c-IAP1: Cellular inhibitor of apoptosis 1
EMT: Epithelial–mesenchymal transition
ZEB2: Zinc finger E-box-binding homeobox 2
MTA1: Metastasis associated 1 protein
circTRIM33-12: Tripartite motifs containing 33-derived circRNA
FGFR1: Fibroblast growth factor receptor
MAGE: Melanoma-associated antigen
FBP1: Fructose-1,6-bisphosphatase 1
FSP27: Fat-specific protein 27
TAK1: TGF-β-activated kinase 1
CSCs: Liver cancer stem cells
IRF1: IFN regulatory factor-1
ISG: Interferon-stimulated gene
DEN: Diethylnitrosamine
RASSF6: Ras association domain-containing protein 6
ATO: Arsenic trioxide
ALDH3A1: Aldehyde dehydrogenase 3 family member A1
UPS: Ubiquitin system
PROTACs: Proteolysis-targeting chimeras
USP15: Ubiquitin-specific protease 15
