# Supplementary figures and images for: TRIM proteins in hepatocellular carcinoma
Source: J Biomed Sci. 2022 Sep 13;29:69. doi: 10.1186/s12929-022-00854-7 (PMC9469581; doi:10.1186/s12929-022-00854-7)

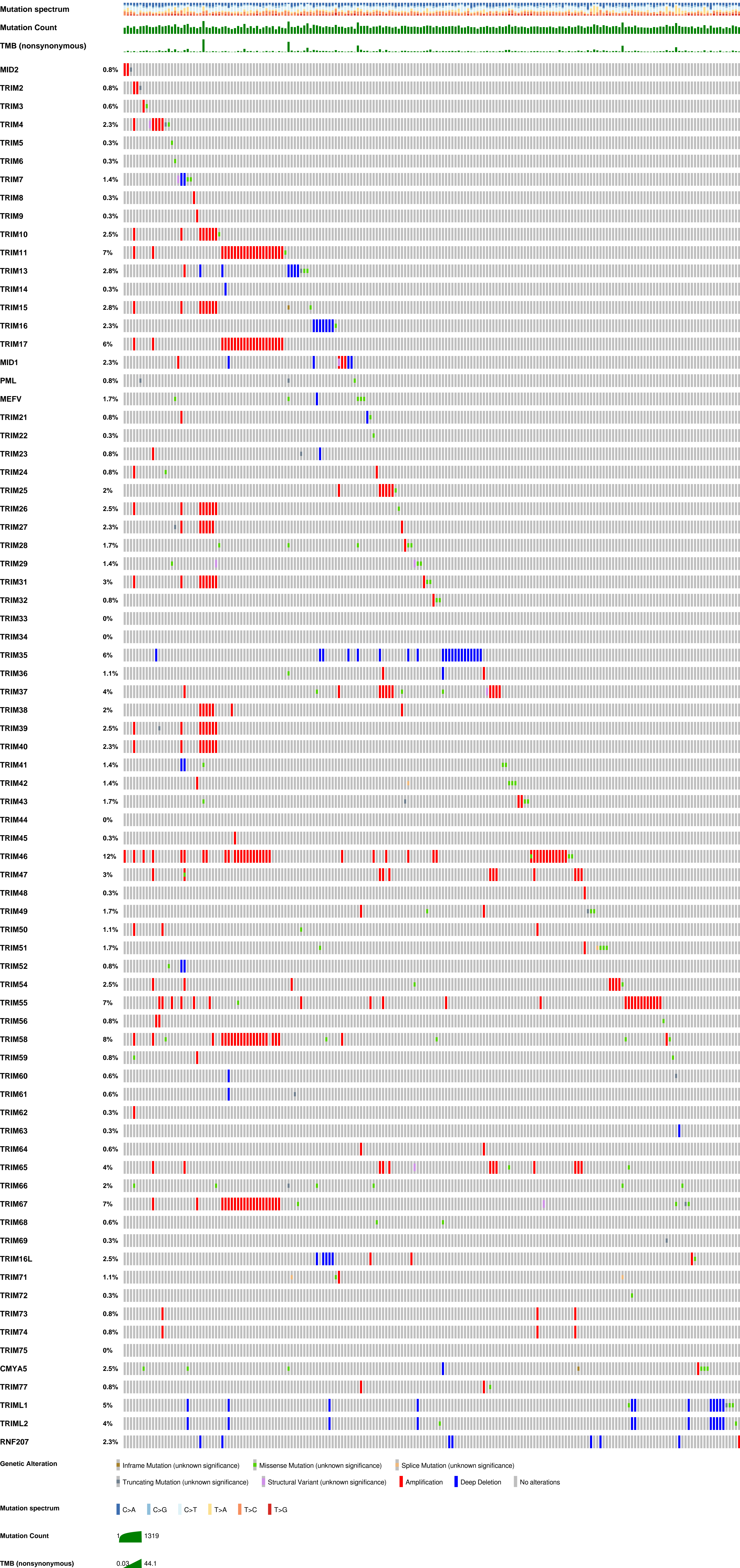

Supplement: Supplementary file 1 — Additional file 1: Fig. S1. Oncoprint plot of all somatic mutations of TRIM proteins in TCGA-LIHC using cbioportal database. [file 12929_2022_854_MOESM1_ESM.pdf]

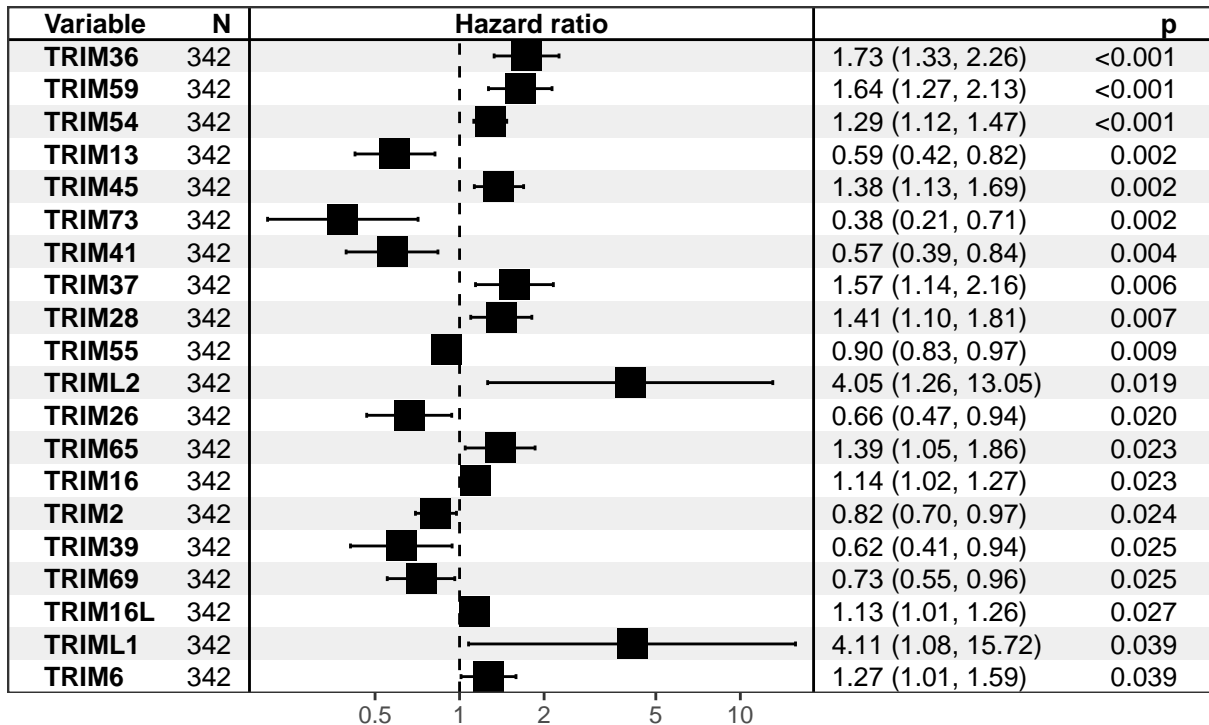

Supplement: Supplementary file 3 — Additional file 3: Fig. S2. A univariate cox analysis of every 75 TRIM proteins based on TCGA-LIHC dataset with p < 0.05. [file 12929_2022_854_MOESM3_ESM.pdf]
